# Distinctions in Breast Tumor Recurrence Patterns Post-Therapy among Racially Distinct Populations

**DOI:** 10.1371/journal.pone.0170095

**Published:** 2017-01-13

**Authors:** Nikita Wright, Jun Xia, Guilherme Cantuaria, Sergey Klimov, Mildred Jones, Pranay Neema, Dora Il’yasova, Uma Krishnamurti, Xiaoxian Li, Michelle D. Reid, Meenakshi Gupta, Padmashree C. G. Rida, Remus Osan, Ritu Aneja

**Affiliations:** 1 Department of Biology, Georgia State University, Atlanta, Georgia, United States of America; 2 Department of Mathematics and Statistics, Georgia State University, Atlanta, Georgia, United States of America; 3 Department of Gynecologic Oncology, Northside Hospital Cancer Institute, Atlanta, Georgia, United States of America; 4 Department of Pathology Oncology Analytics, Northside Hospital Cancer Institute, Atlanta, GA, United States of America; 5 School of Public Health, Georgia State University, Atlanta, Georgia, United States of America; 6 Department of Pathology and Laboratory Medicine, Emory University, Atlanta, Georgia, United States of America; 7 Clinical Pathology and Anatomic Pathology, West Georgia Hospitals, LaGrange, Georgia, United States of America; University of North Carolina at Chapel Hill School of Medicine, UNITED STATES

## Abstract

**Background:**

Clinical studies have revealed a higher risk of breast tumor recurrence in African-American (AA) patients compared to European-American (EA) patients, contributing to the alarming inequality in clinical outcomes among the ethnic groups. However, distinctions in recurrence patterns upon receiving hormone, radiation, and/or chemotherapy between the races remain poorly characterized.

**Methods:**

We compared patterns and rates (per 1000 cancer patients per 1 year) of recurrence following each form of treatment between AA (n = 1850) and EA breast cancer patients (n = 7931) from a cohort of patients (n = 10504) treated between 2005–2015 at Northside Hospital in Atlanta, GA.

**Results:**

Among patients who received any combination of adjuvant therapy, AA displayed higher overall rates of recurrence than EA (p = 0.015; HR: 1.699; CI: 1.108–2.606). Furthermore, recurrence rates were higher in AA than EA among stage I (p = 0.031; HR: 1.736; CI: 1.052–2.864) and T1 classified patients (p = 0.003; HR: 2.009; CI: 1.263–3.197). Interestingly, among patients who received neoadjuvant chemotherapy, AA displayed higher rates of local recurrence than EA (p = 0.024; HR: 7.134; CI: 1.295–39.313).

**Conclusion:**

Our analysis revealed higher incidence rates of recurrence in AA compared to EA among patients that received any combination of adjuvant therapy. Moreover, our data demonstrates an increased risk of tumor recurrence in AA than EA among patients diagnosed with minimally invasive disease. This is the first clinical study to suggest that neoadjuvant chemotherapy improves breast cancer recurrence rates and patterns in AA.

## Introduction

The significant divide in breast cancer mortality between African-American (AA) and European-American (EA) patients remains a challenge for clinicians. Despite a similar number of reported incidences of breast cancer among AA and EA women, AAs experience notably higher severity in clinical outcomes and exhibit a 40% higher death rate than EAs among premenopausal and menopausal breast cancer patients [1–3]. Recurrent breast cancer has impeded successful management of the disease for decades and is one of the primary factors for this racial division in prognosis [4]. Statistics demonstrate that approximately 40% of all breast cancer survivors will experience a recurrence episode during their lifetime, which has been suggested to play a principal role in breast cancer mortality [4, 5]. Clinical studies have revealed a higher risk of recurrence in AA compared to EA, presumably contributing to the inequality in clinical outcomes among the ethnic groups [1]. This statistic has provided an impetus for clinicians to devise and implement robust prognosticative measures to preclude the tumor returning in AA breast cancer patients. However, distinctions in recurrence rates and patterns following various forms of treatment between the races have not been thoroughly evaluated. This warrants more investigation to potentially attenuate the observed racial disparity in recurrence in the clinic. Hence, we conducted a large institutional study based in Atlanta, Georgia, in which we analyzed rates and patterns of tumor recurrence post hormone, radiation, and chemotherapy among AA and EA breast cancer patients. This retrospective clinical study uncovered previously unrecognized distinctions in recurrence patterns following each conventional form of treatment among racially distinct breast populations and may impart valuable clinical insight into preclusive measures for mitigating the ethnic disparity in breast tumor recurrence.

## Materials and Methods

### Study cohort

In this study, a large cohort of breast cancer patients treated at Northside Hospital (NH) in Atlanta, Georgia from 2005 to 2015, were examined. We received approval and permission by the institutional review board at Northside Hospital to access patient clinico-pathological information used in this study and have a written human subjects assurance on file. The demographics and clinico-pathological characteristics of each patient were recorded to generate a database of 10,504 patients. Patient demographic information recorded in the database included age at time of diagnosis and ethnicity. Age at diagnosis among patients was divided into three subgroups, comprised of patients below the age of 48 (premenopausal), over the age of 55 (postmenopausal), and in between (perimenopausal), to precisely describe menopausal status. The races of patients in the database were primarily comprised of African-Americans (AA) and European-Americans (EA). The “unknown/others” subcategory denote patients of all other ethnicities (excluding AA and EA) and patients lacking race information. Ethnicity was self-reported by patients. Breast tumor characteristics that were recorded for each patient consisted of nuclear grade, Nottingham (NGH) grade, stage, nodal status, T (primary tumor), N (lymph node metastasis) and M (distant metastasis) classifications. The 7^th^ edition of the American Joint Committee on Cancer (AJCC)/Union for International Cancer Control (UICC) TNM Classification and Stage groupings for breast carcinoma was used in this article. All patient treatments were recorded, including chemotherapy, hormone, and radiation therapy. Patients that underwent chemotherapy were subcategorized into neoadjuvant and adjuvant depending on the timing of treatment. Additionally, any combination of hormone, radiation, and chemotherapy that patients received was labeled as a combination of adjuvant therapies. Follow up data was collected to determine breast cancer recurrence episodes, as well as the site of the tumor recurrence, such as local, regional or distant sites. Local recurrences comprise the tumor returning to the primary site. Regional recurrence encompasses the reoccurrence of the breast tumor in adjacent lymph nodes. Distant recurrences involve reappearance of the tumors in remote organs such as distant lymph nodes, bone, liver or others.

### Follow-up

Both follow-up of patients and initial diagnosis occurred between the years of 2005 and 2015. Initial diagnosis dates as well as treatment start and completion dates for any therapies were documented. Dates of last contact for all patients were recorded. Survival status (alive/dead) was reported for each patient along with survival time. Dates of first recurrence were noted. February 19, 2015 was the final follow-up for the last patient seen.

### Statistical analysis

A significance level of 0.05 and 95% confidence intervals were selected for all analyses. Sample sizes were based on the available patients that comprised each category in the NH database and not power analysis. Chi-square tests were performed to examine significant differences in clinico-pathological characteristics, therapy administration, and recurrence characteristics between recurrence and non-recurrence patients as well as between AA and EA breast cancer patients. Recurrence rates were calculated as per 1000 person-years (incidence rate) from date of diagnosis until first incidence of the tumor returning over a 10-year period irrespective of specific treatment and for each form of treatment administered. Recurrence was determined as tumor(s) that developed in the same site as the primary tumor or in another local or distant site after the patient went into remission. Test statistics were computed using MATLAB (MATLAB and Statistics Toolbox Release 2015a, The MathWorks, Inc., Natick, Massachusetts, United States) program and 1-tailed univariate p-values were reported. One-tailed analysis was preferred over two-tailed for this particular study to adequately reflect the presumption that treatment is expected to improve patient outcome. Multivariate Cox proportional hazard models were computed to determine significant differences in recurrence rates and patterns between the racial groups [6, 7]. These statistical models were additionally modified to control for variables of age, grade, and stage. The Kaplan Meier analysis was conducted in SAS 9.4 program to estimate survival function for AA and EA with recurrent disease over a 10-year period from time of first tumor reappearance until death or end of follow-up. A log-rank test was conducted to evaluate significance level for between-race differences in survival. Finally, a t-test was used to compare mean time from first recorded tumor reocurrence event until death among patients with distant recurrence.

## Results

### Clinico-pathological characteristics of patients

**Fig 1:**
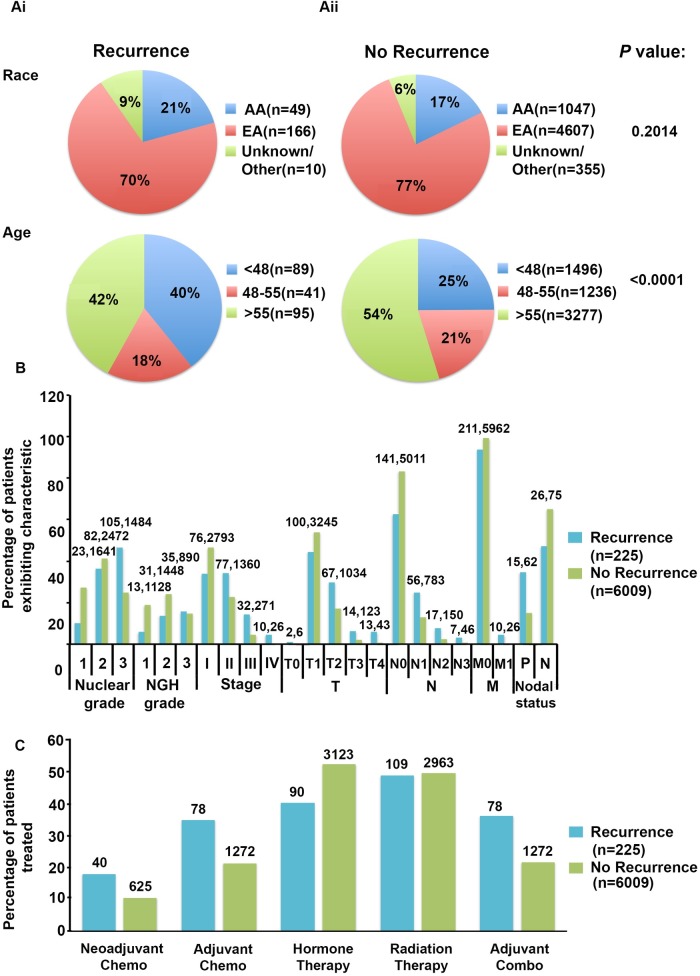
NH demographics, breast clinico-pathological characteristics, and treatment compared between patients with or without tumor recurrence. (**A**) The numbers and percentages of patients displaying demographic and (**B)** breast clinico-pathological characteristics were compared for patients with (n = 225) and without recurrence (n = 6009). (**C**) The numbers and percentages of tumor recurrences for patients whose disease either recurred or did not recur, were compared in patients who underwent each form of conventional breast cancer treatment. Significant differences in all clinico-pathologic characteristics were observed between patients with or without tumor recurrence (p<0.0001), with the exception of nodal status (p = 0.121). Significant differences in clinico-pathological features were also observed between recurrence and non-recurrence patients receiving each form of treatment (p<0.0001), except for radiation therapy (p = 0.065). A chi-square statistical analysis was used to generate p-values in order to determine significant differences in the proportion of patients exhibiting or not exhibiting recurrence in each category. For example, regarding grade, a p value of 0.02 represents a significant difference for the distribution of recurrence and non-recurrence patients across all grades. Please refer to Supplementary Table 1 for details.

The demographics, breast clinico-pathological characteristics, therapies administered and patterns of recurrence among the patients in the cohort are illustrated in Fig 1. From this cohort of 10,504 NH patients, 225 were recorded as having experienced a recurrence episode and 6,009 were determined as displaying no reappearance of the breast tumor. The remaining patients did not have recorded data indicating presence of recurrence or lack thereof. Among patients displaying recurrence, higher risk of tumor reocurrence was more prevalent among younger patients (p<0.0001) (Fig 1A). This result is consistent with previous studies that have observed an association between younger age and increased risk for recurrence [8–10]. Particularly, age under 35 years was able to serve as a successful independent prognostic factor for time to recurrence in a large retrospective study of early-stage breast cancer patients [10]. Furthermore, de la Rochefordiere *et al*. found recurrence rate decreased by 4% with every year of age in premenopausal breast cancer patients [9]. Among patients with no missing recurrence data, approximately 61% of patients who experienced the tumor returning were under the age of 48, compared to only 39% who did not experience any recurrence. Among breast clinico-pathological characteristics, recurrence was significantly more associated with higher nuclear grade, NGH grade, stage, as well as T, N, and M classifications (p<0.0001) (Fig 1B). Moreover, tumor reoccurrence was weakly associated with lymph node metastasis with roughly 35% of patients with recurrence displaying a positive nodal status compared to only 15% of non-recurrence patients (p = 0.121). These results further confirm previous findings of increased risk of recurrence associated with more aggressive tumor characteristics. Particularly, lymph node involvement, larger tumor size, higher grade, lymphovascular invasion, and proliferation markers such as S-phase fraction strongly correlate with increased risk for recurrence [11]. Regarding treatment, there were statistical significant differences in the distribution of recurrence and non-recurrence patients who were administered neoadjuvant and adjuvant chemotherapy, hormone therapy, and a combination of adjuvant therapies (p<0.0001) (Fig 1C). There was a weak statistical significant difference between the proportion of recurrence and non-recurrence patients that received radiation therapy (p = 0.065). Please see S1 Table for details.

### Clinico-pathological characteristics among racially distinct patients

**Fig 2:**
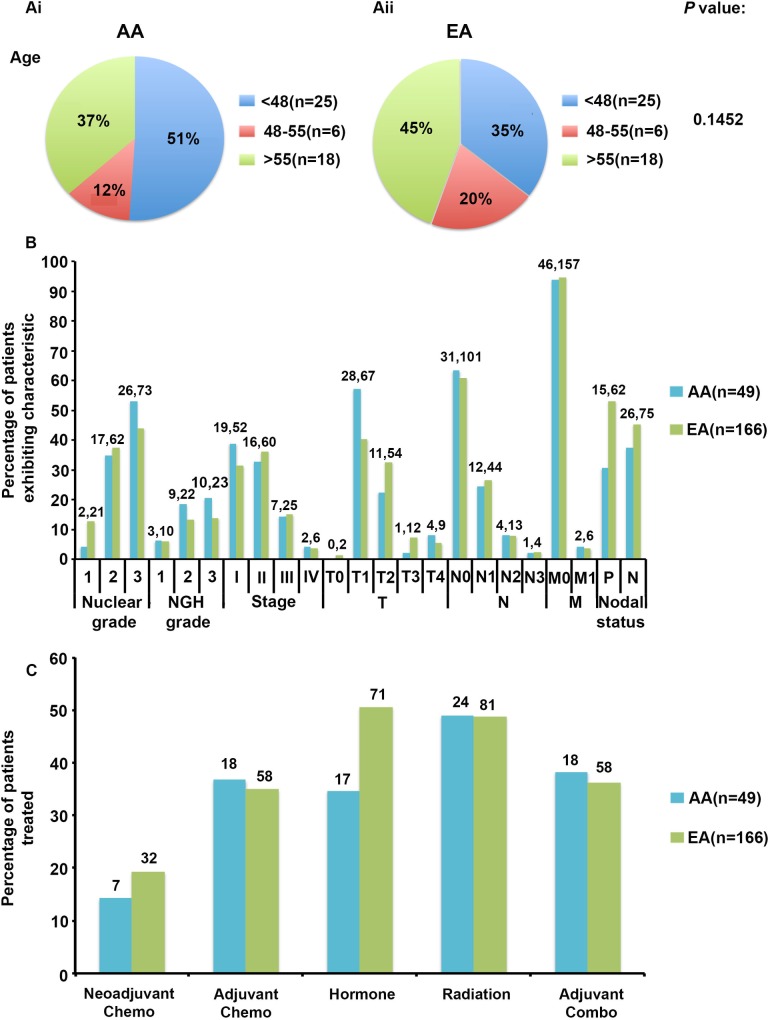
NH demographics, breast, clinico-pathological and treatment compared between AA and EA with tumor recurrence. **(A)** The demographic and **(B)** clinico-pathological characteristics of AA (n = 49) and EA (n = 166) patients with recurrence in the NH cohort were compared. (**C**) Treatment administration was also compared between AA and EA recurrent breast cancer patients. A chi-square analysis was used to determine statistically significant differences in the proportion of AA and EA patients exhibiting each characteristic and undergoing each form of treatment. No statistically significant differences were observed. Please refer to Supplementary Table 2 for details.

Among the NH patients exhibiting recurrence, the demographics, breast clinico-pathological features, and therapies administered were compared between AA (n = 49) and EA (n = 166) as shown in Fig 2. Regarding patient demographic characteristics, a test of hypothesis for population differences revealed a weak statistical significant difference in age at diagnosis between the races (p = 0.145) (Fig 2A). Approximately 51% of AA were diagnosed under the age of 48, compared to only 35% of EA. Roughly 37% of AA were diagnosed over the age of 55, compared to 45% of EA. Furthermore, 20% of EA were diagnosed between 48–55, while only 12% of AA were diagnosed between 48–55. This finding, however, corroborates the well-established finding that AA are diagnosed at a much younger age with breast cancer than EA [12–14]. The median age at diagnosis reported for AA and EA is 54 and 61, respectively [14]. The mean age at diagnosis for AA and EA in our study was 54.569 (CI: 54.284–54.853) and 58.061 (CI: 57.922–58.199), respectively (p<0.001) (S1 Fig). Data from multiple state registries reveal that on average, 12.4% of AA and 5.7% of EA present under the age of 40 [15]. Furthermore, SEER data indicates that AA have an age-specific incidence rate between 30–39 years compared to 40.79 for EA [14]. No statistically significant differences in either clinico-pathological characteristics or treatments between the races were observed, likely owing to a significant reduction in patient numbers after stratification of recurrence patients by race (Fig 2B and 2C). Please consult S2 Table for details.

### Recurrence patterns among racially distinct patients

Recurrence rates and patterns, expressed in terms of incidence rates, were compared broadly (regardless of specific treatment administered) between AA and EA patients (Table 1); unadjusted analyses indicated that AA exhibited higher overall tumor recurrence rates than EA (p = 0.002; HR: 1.676; CI: 1.210–2.323), which further confirms similar previous findings [1,16]. AA also displayed higher rates of distant recurrence than EA (p = 0.023; HR: 1.699; CI: 1.075–2.684. Additionally, AA experienced higher rates of single tumor recurrence episodes than EA (p = 0.003; HR: 1.758; CI: 1.208–2.557) and higher rates of distant recurrence to a single site than EA breast cancer patients (p = 0.012; HR: 1.742; CI: 1.130–2.684). For patients exhibiting multiple recurrences, endpoint was determined as the reappearance of tumors in all observed sites after the first indication of recurrence.

**Table 1:** Broad-spectrum recurrence patterns among racially distinct populations.

	EA		AA		p value; HR (95% CI)	p value; HR (95% CI)
	n	IR	n	IR	Unadjusted Model	Adjusted Model
**Overall**	166	13.44	49	21.77	**0.002; 1.676 (1.210, 2.323)**	0.319; 1.192 (0.844, 1.683)
**Recurrence site**						
Local	48	3.89	12	5.33	0.373; 1.349 (0.698, 2.606)	0.665; 0.857 (0.428, 1.718)
Regional	27	2.19	10	4.44	0.188; 1.701 (0.772, 3.747)	0.151; 1.749 (0.815, 3.752)
Distant	84	6.8	27	12	**0.023; 1.699 (1.075, 2.684)**	0.280; 1.299 (0.809, 2.085)
**Number of recurrences**						
Single	131	10.6	41	18.21	**0.003; 1.758 (1.208, 2.557)**	0.218; 1.287 (0.861, 1.923)
Multiple	35	2.83	8	3.55	0.754; 1.139 (0.505, 2.573)	0.315; 0.652 (0.283, 1.503)
**Distant recurrence**						
Single site	73	5.91	24	10.66	**0.012; 1.742 (1.130, 2.684)**	0.451; 1.220 (0.728, 2.043)
Multiple sites	11	0.89	3	1.33	0.492; 1.566 (0.436, 5.625)	0.617; 0.672 (0.142, 3.187)

Abbreviations: AA, African-American; EA, European-American; HR, hazard rate; IR, incidence rate (1000 person-years); CI, confidence interval. Adjusted Cox hazard model variables: age at diagnosis, grade (1,2,3), and stage (I,II,III,IV).

*P values were calculated using the student t-test.

### Recurrence patterns among racially distinct patients following each form of treatment

Incidence rates and patterns of recurrence were compared between AA and EA after they received hormone, radiation, chemotherapy, and/or any combination of adjuvant therapy to determine distinctions in recurrence patterns between therapies among the racial groups (Table 2). AA exhibited unadjusted higher rates of recurrence (p = 0.041; HR: 1.612; CI: 1.021–2.545) and a trend towards higher incidence of distant recurrence than EA post radiation therapy (p = 0.065; HR: 1.732; CI: 0.967–3.100). The same trend of higher overall and distant recurrence was observed among recurrent patients who received hormone therapy and any combination of adjuvant therapies. Among patients who underwent hormone therapy, AA displayed stronger overall tendencies than EA to suffer from recurrence (p = 0.112; HR: 1.541; CI: 0.906–2.623) and distant recurrence (p = 0.123; HR: 1.692; CI: 0.868–3.301). Following any combination of adjuvant therapy, AA displayed higher recurrence rates than EA after adjusting for age, grade, and stage (p = 0.015; HR: 1.699; CI: 1.108–2.606). Moreover, unadjusted analyses reveal AA displayed higher rates of distant recurrence than EA (p = 0.003; HR: 2.164; 1.290–3.629) as well as stronger tendencies toward regional recurrence (p = 0.104; HR: 2.043; CI: 0.863–4.837) after receiving any combination of adjuvant therapy.

**Table 2:** Recurrence rates and patterns after receiving any form of treatment among racially distinct populations.

	EA		AA		p value; HR (95% CI)	p value; HR (95% CI)
Treatment	n	IR	n	IR	Unadjusted model	Adjusted model
**Chemotherapy**						
Overall	85	22.73	23	26.66	0.466; 1.181 (0.755, 1.846)	0.807; 0.943 (0.587, 1.514)
Local	13	3.48	7	7.46	0.125; 2.053 (0.818, 5.151)	0.394; 1.548 (0.567, 4.226)
Regional	20	5.35	6	6.4	0.594; 1.284 (0.512, 3.219)	0.749; 1.169 (0.450, 3.041)
Distant	50	13.37	12	12.79	0.832; 0.934 (0.498, 1.751)	0.613; 0.840 (0.426,1.653)
**Neoadjuvant chemotherapy**						
Overall	32	28.95	6	19.77	0.373; 0.673 (0.281, 1.609)	0.409; 0.690 (0.286, 1.664)
Local	2	1.81	4	13.18	**0.026; 6.857 (1.256, 37.447)**	**0.024; 7.134 (1.295, 39.313)**
Regional	7	6.33	0	0	N/A	N/A
Distant	23	20.81	2	6.59	0.112; 0.310 (0.073, 1.315)	0.136; 0.332 (0.078, 1.417)
**Adjuvant chemotherapy**						
Overall	57	20.96	18	26.16	0.405; 1.253 (0.737, 2.130)	0.891; 1.039 (0.603, 1.788)
Local	12	4.41	3	4.36	0.865; 0.897 (0.255, 3.153)	0.500; 0.645 (0.181, 2.303)
Regional	15	5.52	6	8.72	0.333; 1.598 (0.619, 4.125)	0.843; 1.102 (0.421, 2.885)
Distant	30	11.03	9	13.08	0.664; 1.179 (0.561, 2.480)	0.100; 1.000 (0.463, 2.159)
**Hormone therapy**						
Overall	69	10.45	17	15.94	0.112; 1.541 (0.906, 2.623)	0.949; 1.020 (0.568, 1.830)
Local	15	2.27	2	1.87	0.676; 0.731 (0.169, 3.172)	0.290; 0.332 (0.043, 2.558)
Regional	14	2.12	4	3.75	0.369; 1.654 (0.552, 4.959)	0.580; 1.380 (0.442, 4.305)
Distant	40	6.06	11	10.31	0.123; 1.692 (0.868, 3.301)	0.482; 1.307 (0.619, 2.757)
**Radiation therapy**						
Overall	79	12.62	23	19.34	**0.041; 1.612 (1.021, 2.545)**	0.986; 1.004 (0.609, 1.658)
Local	22	3.52	6	5.04	0.450; 1.414 (0.575, 3.475)	0.689; 0.816 (0.302, 2.205)
Regional	10	1.6	3	2.52	0.532; 1.503 (0.419, 5.392)	0.736; 1.264 (0.324, 4.490)
Distant	47	7.51	15	12.61	0.065; 1.732 (0.967, 3.100)	0.810; 1.083 (0.568, 2.063)
**Adjuvant radiation, hormone, and chemotherapy**						
Overall	101	11.94	30	19.63	**0.013; 1.678 (1.115, 2.524)**	**0.015; 1.699 (1.108, 2.606)**
Local	31	3.66	3	1.96	0.279; 0.520 (0.159, 1.698)	0.145; 0.405 (0.121, 1.364)
Regional	19	2.25	7	4.58	0.104; 2.043 (0.863, 4.837)	0.558; 1.310 (0.531, 3.230)
Distant	51	6.03	20	13.09	**0.003; 2.164 (1.290, 3.629)**	0.101; 1.607 (0.912, 2.833)

Abbreviations: AA, African-American; EA, European-American; HR, hazard rate; IR, incidence rate (1000 person-years); CI, confidence interval. Adjusted Cox hazard model variables: age at diagnosis, grade (1,2,3), and stage (I,II,III,IV).

*P values were calculated using the student t-test.

Quite interestingly however, among patients with recurrence that received neoadjuvant chemotherapy, this trend was reversed. AA displayed lower tendencies toward tumor recurrence than EA patients. Furthermore, AA displayed lower tendencies toward regional and distant tumor recurrence (p = 0.112; HR: 0.310; CI: 0.073–1.315) than EA patients. Moreover, AA displayed higher rates of local recurrence than EA after controlling for age, grade, and stage (p = 0.024; HR: 7.134; CI: 1.295–39.313). These results suggest that aggressive recurrence rates and patterns may be attenuated in AA patients who received neoadjuvant chemotherapy. Additional studies with larger numbers of patients with recurrence that received neoadjuvant chemotherapy could further clarify the significance of this trend.

### Recurrence rates among racially distinct breast cancer patients in different stages

Overall incidence rates of recurrence were compared between AA and EA in both early (I–II) and late stage (III-IV) breast cancer patients (Table 3). Our data revealed that AA displayed higher recurrence rates than EA among stage I patients (p = 0.001; HR: 2.165; CI: 1.348–3.476), even after adjusting for age, grade, and stage (p = 0.031; HR: 1.736; CI: 1.052–2.864). Among early stage (I-II) patients, AA also exhibited higher recurrence rates than EA (p = 0.002; HR: 1.793; CI: 1.252–2.567); this trend persisted after controlling for age, grade, and stage (p = 0.131; HR: 1.339; CI: 0.917–1.956). Furthermore, AA displayed higher recurrence rates than EA among T1 classified patients, irrespective of age, grade, and stage (p = 0.003; HR: 2.009; CI: 1.263–3.197). Moreover, unadjusted models reveal that AA displayed higher rates of recurrence than EA among N0 (p = 0.005; HR: 1.777; CI: 1.186–2.661) and M0 (p = 0.002; HR: 1.682; CI: 1.210–2.338) classified patients. However, rates of recurrence were not significantly higher in AA as compared to EA among late stage patients. Thus, these results suggest that AA are at higher risk than EA for tumor the tumor recurrence among patients with non-invasive or minimally invasive breast cancer.

**Table 3:** Overall recurrence rates among racially distinct staged breast cancer patients.

	EA		AA		p value; HR (95% CI)	p value; HR (95% CI)
	n	IR	n	IR	Unadjusted Model	Adjusted Model
**Grouped stage**						
Early (I-II)	130	11.14	39	19.08	**0.002; 1.793 (1.252, 2.567)**	0.131; 1.339 (0.917, 1.956)
Late (III-IV)	31	55.17	9	50.65	0.857; 0.934 (0.445, 1.962)	0.637; 0.823 (0.366, 1.850)
**Individual Stage**						
I	70	7.76	23	15.84	**0.001; 2.165 (1.348, 3.476)**	**0.031; 1.736 (1.052, 2.864)**
II	60	22.67	16	27.02	0.447; 1.239 (0.713, 2.154)	0.823; 0.936 (0.523, 1.674)
III	25	48.01	7	45.01	0.902; 0.949 (0.410, 2.195)	0.590; 0.774 (0.306, 1.959)
IV	6	145.8	2	90.29	0.822; 0.832 (0.167, 4.152)	0.967; 0.964 (0.168, 5.518)
**TNM Staging**						
**T**						
T0	2	130.83	0	N/A	N/A	N/A
T1	67	9.75	28	25.63	**<0.0001; 2.776 (1.781, 4.326)**	**0.003; 2.009 (1.263, 3.197)**
T2	54	28.48	11	22.3	0.504; 0.801 (0.419, 1.534)	0.215; 0.647 (0.325, 1.287)
T3	12	49.67	1	13.31	0.215; 0.275 (0.035, 2.115)	0.161; 0.228 (0.029, 1.796)
T4	9	106.73	4	121.36	0.680; 1.282 (0.394, 4.173)	0.983; 1.015 (0.241, 4.270)
**N**						
N0	101	9.81	31	16.89	**0.005; 1.777 (1.186, 2.661)**	0.211; 1.319 (0.854, 2.037)
N1	44	27.05	12	39.2	0.201; 1.518 (0.801, 2.877)	0.828; 1.079 (0.545, 2.136)
N2	13	46.45	4	53.23	0.744; 1.207 (0.391, 3.719)	0.965; 0.970 (0.258, 3.646)
N3	5	55.06	1	43.27	0.742; 0.697 (0.081, 5.970)	0.974; 0.962 (0.095, 9.711)
**M**						
M0	157	12.82	46	20.8	**0.002; 1.682 (1.210, 2.338)**	0.288; 1.210 (0.851, 1.721)
M1	6	145.8	2	90.29	0.822; 0.832 (0.167, 4.152)	0.967; 0.964 (0.168, 5.518)

Abbreviations: AA, African-American; EA, European-American; HR, hazard rate; IR, incidence rate (1000 person-years); CI, confidence interval. Adjusted Cox hazard model variables: age at diagnosis, grade (1,2,3), and stage (I,II,III,IV).

*P values were calculated using the student t-test.

### Survival outcomes among racially distinct patients displaying recurrence

**Fig 3:**
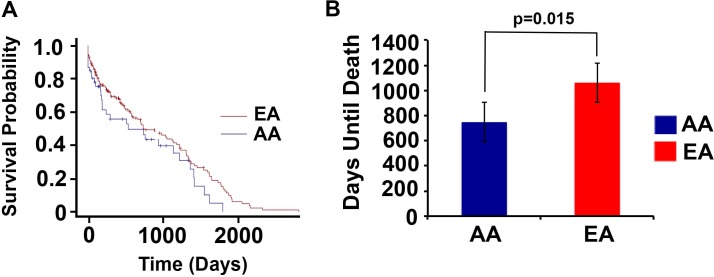
AA exhibit lower survival duration than EA among recurrent breast cancer patients. (**A**) Survival time from first recurrence episode until death was compared between AA and EA breast cancer patients. Log-rank analysis was conducted to determine statistical differences between the racial groups. AA exhibited a non-significant lower survival time than EA (p = 0.231). (**B**) The mean time (days) until death was compared between AA and EA breast cancer patients displaying distant recurrence. AA died notably sooner than EA patients (p = 0.015). A t-test was performed to determine significant differences between the racial groups.

Survival duration after initial recorded recurrence was compared between AA and EA patients to determine if there are differences in time until death after tumor(s) reappear between AA and EA breast cancer patients (Fig 3). These findings may further illuminate how recurrence is driving the racial disparity in survival outcomes. AA exhibited a non-statistically significant trend toward shorter survival time than EA after experiencing their first episode of recurrence (p = 0.231) (Fig 3A**).** The average time until death was compared between EA and AA patients who experienced distant recurrences (Fig 3B). Interestingly, AA and EA patients exhibiting distant recurrence were comprised of similar percentages of alive patients, however AA (n = 26) died considerably sooner than EA (n = 80) (p = 0.015). More precisely, AA patients who experienced distant recurrences died approximately one year earlier than EA distant recurrent patients.

## Discussion

This clinical study is the first extensive investigation into the rates and patterns of tumor recurrence in breast cancer patients following conventional treatments among racially distinct populations. Our study has revealed notable distinctions in recurrence patterns among EA and AA patients. First, AA displayed considerably higher rates of recurrence than EA (Tables 1 and 2). Second, we observed higher severity in recurrence patterns displayed by AA for whom we discerned stronger trends in AA of the tumor recurrence to regional and distant sites (Tables 1 and 2). This trend was evident after patients received radiation, hormone, and any combination of adjuvant therapies. Overall, these observed trends were quite significant since local recurrence tends to elicit a more favorable clinical prognosis compared to distant recurrence, while the latter trends type precedes a poorer clinical prognosis. Triple negative breast cancer (TNBC) patients have been shown to display an increased risk for recurrence and particularly for tumor reocurrence to distant sites, while non-TNBC patients exhibit higher trends of the tumor reappearing in local sites [1,17]. These findings parallel our observations of an increased risk of overall and especially distant recurrence in AA, as well as an increased risk of local recurrences in EA. This tendency reflects the well-reported higher incidence of TNBC phenotypes in AA patients and a higher prevalence of non-TNBC subtypes in EA patients [18,19]. Furthermore, we observed a trend of a higher number of recurrence episodes in AA compared to EA. Additionally, we discerned stronger inclinations of distant recurrence to multiple organs in AA compared to EA. These observed aggressive recurrence patterns reveal that AA patients exhibit an increased prospect of a poor clinical prognosis, theoretically contributing to their higher mortality rates than EA patients. Recurrence rates were also found to be higher in AA than EA among early stage, minimally invasive breast cancer patients (Table 3). This data presents an intriguing paradox as advanced stage upon diagnosis is typically associated with increased risk for recurrence [19–22]. Thus, these findings suggest that AA patients of all clinical stages should be closely evaluated for the prospect of tumor recurrence. Neoadjuvant chemotherapy seemed to reverse these observed recurrence trends (Table 2). Among patients who received neoadjuvant chemotherapy, AA displayed a lower rate of recurrence than EA; however due to a low number of recorded patients that received neoadjuvant chemotherapy, statistical significance was diminished. In addition, higher incidences of aggressive recurrence patterns in AA were notably attenuated after these patients underwent neoadjuvant chemotherapy. This data suggest preoperative chemotherapy may reduce the severity of recurrence rates and patterns in AA patients. This study suggests that neoadjuvant chemotherapy should be recommended for AA patients who are at higher risk for developing recurrence. A recent clinical study reported that in fact, neoadjuvant chemotherapy is administered more frequently to AA than EA patients likely as a result of their higher prevalence of advanced stage, grade, and triple negative receptor status upon presentation [23].

Rigorous surveillance for tumor recurrence is conceivably crucial to mitigate the elevated risk of recurrent breast cancer in AA patients. Owing to their robust prognostic value, the extent of lymph node involvement and tumor size warrant stringent evaluation upon diagnosis and serve as principal prognostic factors in assessing breast cancer recurrence proclivities in AA patients [1]. Established supplementary prognostic factors often considered by clinicians, such as higher stage and grade upon initial diagnosis, lymphatic and vascular invasion, premenopausal status, and a TNBC phenotype, also merit thorough scrutiny for AA breast cancer patients [18,19–22,24–26]. In addition, age has been shown to provide strong prognostic value. Our dataset revealed a higher trend toward diagnosis at a younger age in AA compared to EA patients, which has been supported by several previous studies for both the onset of breast cancer as well as its reappearance after remission in AA patients. Differences in tumor biology between the races have been suggested to play a significant role in the disparity in age at diagnosis. A recent genome-wide study identified a genetic variant in the *LOC643714 gene* in AA that is associated with a 23% increased risk for developing breast cancer [27]. Studies have also identified a *Msp1* single nucleotide polymorphism in the CYP1A1 gene, a gene involved in estrogen metabolism and action, to be associated with breast cancer in AA but not in EA and the HSD17B1 312 Gly allele to be specifically associated with increased risk in AA for premenopausal breast cancer [28,29]. Also, genetic abnormalities associated with increased proliferation in breast cancer such as TNBC tumors, higher grade, higher expression of cell cycle regulatory proteins such as cyclin E, and increased incidence of mutations in tumor suppressor genes such as *p53*, *RASSF1A*, *RARβ*, and *HIN-1* are known to accelerate the development of breast tumors and are more prevalent among AA compared to EA [14]. Non-biological factors have also been suggested to contribute to the earlier age of onset of breast cancer in AA such as higher parity, younger age at giving birth, full term pregnancies, lower breast-feeding rates, and higher waist-to-hip ratio [14]. Age was also able to serve as a prognosticator for recurrence in our study as well as in other studies, in which younger age was significantly associated with an increased risk. Hence, these findings reinforce age as a critical prognostic factor and urge the need to review guidelines related to the minimum age for screening for AA women. Additional studies correlating prognostic factors such as age with more markers of aggressive tumor biology may be helpful in uncovering molecular mechanisms underlying the more aggressive phenotypes and poorer clinical prognoses in AA patients, and to lay the grounds for developing promising targeted therapeutics for this racial group.

Although prior clinical studies have exposed disparities in recurrence risk among EA and AA, this study is one of the first to uncover distinctions in rates and patterns of tumor recurrences following conventional forms of breast cancer treatments among the racial groups, and thus highlights the need for further investigation and surveillance. Our comprehensive analysis has also illuminated previously unrecognized differences in the rates and patterns of recurrence post-chemotherapy among racially distinct populations by suggesting that AA respond better to neoadjuvant chemotherapy. Additionally, no study has yet elucidated the significantly higher risk for recurrence among early stage AA patients. However, because our study only provides information on a recurrence event following initial documented surgical or therapeutic intervention, the influence of biological, environmental, socioeconomic, and screening factors on the variation in recurrence rates and patterns between the ethnic groups remains unclear. Both biological and environmental factors have been suggested to play a critical role in the divergence in recurrence rates between the racial groups [1,3,12,30]. Racial differences in the prevalence of *TP53* gene mutations, PAM50 basal subtype, and TNBC diagnoses have been suggested to influence the racial disparity in breast tumor recurrence [1]. Socioeconomic deprivation has been associated with decreased screenings and lack of timely detection of breast cancer, which may adversely influence the risk and severity of tumor recurrence [30–33]. Socioeconomically deprived and ethnic minority women were more likely to be diagnosed with late stage breast cancer than their counterparts [31,32]. A later stage upon presentation may increase the likelihood for these patients to develop tumor recurrence. Therefore, these factors warrant investigation as potential underlying drivers of the observed ethnic disparities in recurrence rates and patterns in future studies. Furthermore, our study primarily considers the evolved primary tumor that has already acquired metastatic and recurrent characteristics and thus does not take into an account the mutational shift and acquisition of aggressive phenotypes in nascent tumors. Deeper examination of these influential factors should be conducted in future studies to acquire a thorough understanding and explanation for the evident racial disparity in recurrence rates and patterns.

Nonetheless, our study further advocates that race should be considered among the crucial risk factors in the clinic for recurrence. Awareness of the higher rate of tumor recurrences in AA may compel clinicians to consider race as a critical factor in evaluating the prospect of the cancer returning after patients enter remission, and allow this factor to play a major role in treatment decisions. Hereinafter, enriched comprehensive screening programs and tailored treatment plans may be imperative to impede augmented risk of tumor reocurrences and aggressive recurrence patterns in AA patients that may be reinforcing their poor clinical outcomes.

## Supporting Information

S1 Fig Age at presentation among AA and EA breast cancer patients at NH. The distribution of age at diagnosis compared between AA and EA breast cancer patients at NH. The mean age at presentation for AA and EA patients is 54.569 (CI: 54.284–54.853) and 58.061 (CI: 57.922–58.199), respectively (p<0.001).
(TIF)

S1 Table NH breast cancer patients’ demographics, clinico-pathological and treatment characteristics compared between patients with or without tumor recurrence. (DOCX)

S2 Table NH breast cancer patients’ demographics, clinico-pathological and treatment characteristics compared between AA and EA with recurrence. (DOCX)
