# Supplementary figures and images for: Distinctions in Breast Tumor Recurrence Patterns Post-Therapy among Racially Distinct Populations
Source: PLoS One. 2017 Jan 13;12(1):e0170095. doi: 10.1371/journal.pone.0170095 (PMC5234824; doi:10.1371/journal.pone.0170095)

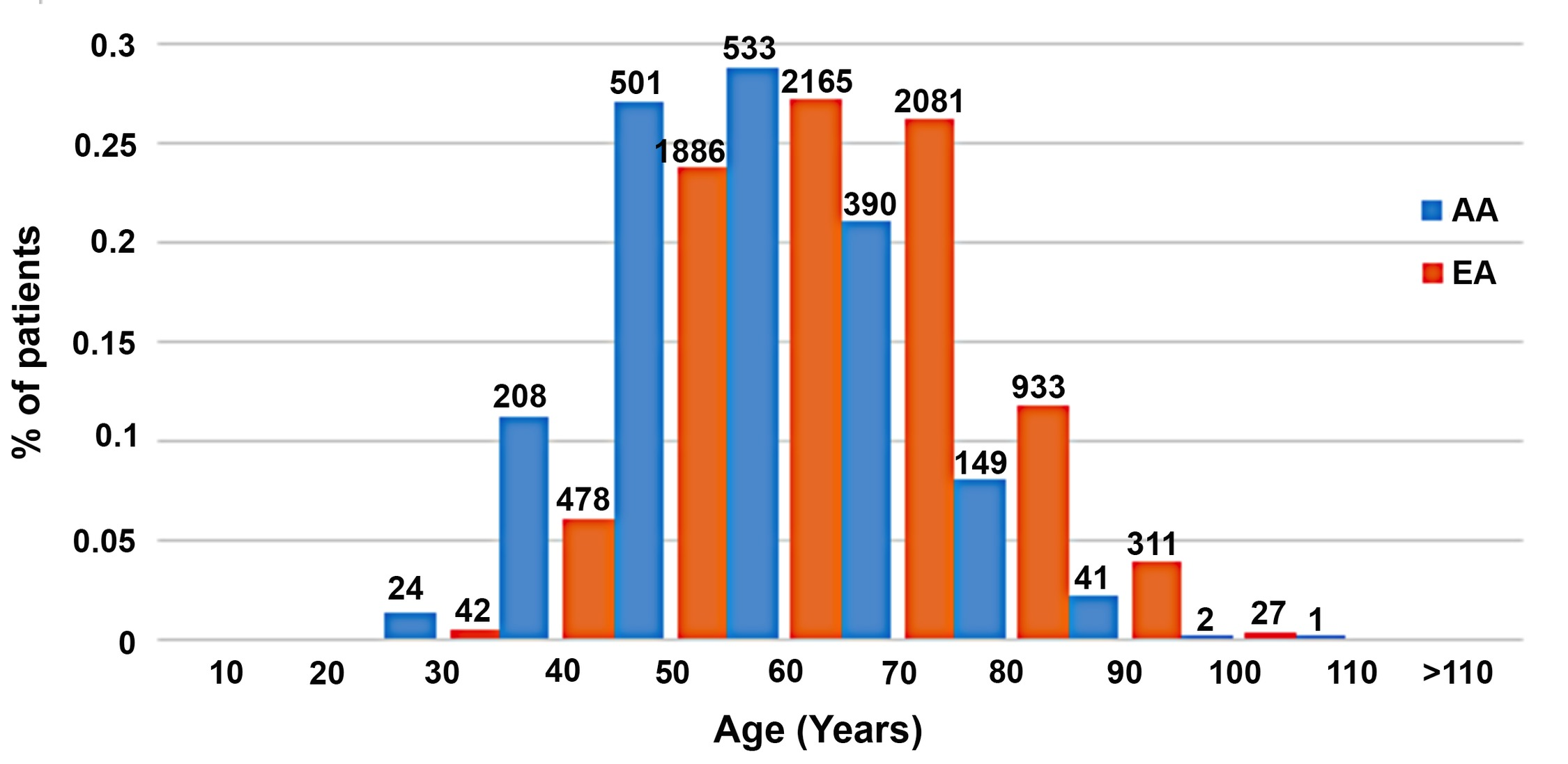

Supplement: S1 Fig — The distribution of age at diagnosis compared between AA and EA breast cancer patients at NH. The mean age at presentation for AA and EA patients is 54.569 (CI: 54.284–54.853) and 58.061 (CI: 57.922–58.199), respectively (p<0.001). (TIF) [file pone.0170095.s001.tif]
