# Supplementary material for: Distinctions in Breast Tumor Recurrence Patterns Post-Therapy among Racially Distinct Populations
Source: PLoS One. 2017 Jan 13;12(1):e0170095. doi: 10.1371/journal.pone.0170095 (PMC5234824; doi:10.1371/journal.pone.0170095)
Supplement: S1 Table — (DOCX) [file pone.0170095.s002.docx]

**S1 Table. NH breast cancer patients’ demographics, clinico-pathological and treatment characteristics compared between patients with or without tumor recurrence.**

|  | **Recurrence** | | **No recurrence** | |  |
| --- | --- | --- | --- | --- | --- |
| **Clinical characteristic** | **n=225** | **%** | **n=6009** | **%** | ***P*** |
| **Ethnicity** |  |  |  |  |  |
| AA | 49 | 21.78 | 1047 | 17.42 | 0.2014 |
| EA | 166 | 73.78 | 4607 | 76.67 |  |
| Unknown/Other | 10 | 4.44 | 355 | 5.91 |  |
| **Menopausal status** |  |  |  |  |  |
| <48 | 89 | 39.56 | 1496.00 | 24.90 | <0.0001 |
| 48-55 | 41 | 18.22 | 1236.00 | 20.57 |  |
| >55 | 95 | 42.22 | 3277 | 54.53 |  |
| **Nuclear grade** |  |  |  |  |  |
| 1 | 23 | 10.22 | 1641.00 | 27.31 | <0.0001 |
| 2 | 82 | 36.44 | 2472.00 | 41.14 |  |
| 3 | 105 | 46.67 | 1484.00 | 24.70 |  |
| Missing | 15 | 6.67 | 412.00 | 6.86 |  |
| **Nottingham grade** |  |  |  |  |  |
| 1 | 13 | 5.78 | 1128.00 | 18.77 | <0.0001 |
| 2 | 31 | 13.78 | 1448.00 | 24.10 |  |
| 3 | 35 | 15.56 | 890.00 | 14.81 |  |
| Missing | 146 | 64.89 | 2543.00 | 42.32 |  |
| **Stage** |  |  |  |  |  |
| 0 | 24 | 10.67 | 1518.00 | 25.26 | <0.0001 |
| I | 76 | 33.78 | 2793.00 | 46.48 |  |
| II | 77 | 34.22 | 1360.00 | 22.63 |  |
| III | 32 | 14.22 | 271.00 | 4.51 |  |
| IV | 10 | 4.44 | 26.00 | 0.43 |  |
| Missing/unknown | 6 | 2.67 | 41.00 | 0.68 |  |
| **Nodal status** |  |  |  |  |  |
| Positive | 78 | 34.67 | 911.00 | 15.16 | 0.121 |
| Negative | 106 | 47.11 | 3920.00 | 65.24 |  |
| Missing/unknown | 41 | 18.22 | 1170.00 | 19.47 |  |
| **TNM Staging** |  |  |  |  |  |
| **T** |  |  |  |  |  |
| T0 | 2 | 0.89 | 6.00 | 0.10 | <0.0001 |
| TX | 2 | 0.89 | 30.00 | 0.50 |  |
| Tis | 24 | 10.67 | 1518.00 | 25.26 |  |
| T1 | 100 | 44.44 | 3245.00 | 54.00 |  |
| T2 | 67 | 29.78 | 1034.00 | 17.21 |  |
| T3 | 14 | 6.22 | 123.00 | 2.05 |  |
| T4 | 13 | 5.78 | 43.00 | 0.72 |  |
| Unknown | 3 | 1.33 | 10.00 | 0.17 |  |
| **N** |  |  |  |  |  |
| N0 | 141 | 62.67 | 5011.00 | 83.39 | <0.0001 |
| NX | 1 | 0.44 | 9.00 | 0.15 |  |
| N1 | 56 | 24.89 | 783.00 | 13.03 |  |
| N2 | 17 | 7.56 | 150.00 | 2.50 |  |
| N3 | 7 | 3.11 | 46.00 | 0.77 |  |
| Unknown | 3 | 1.33 | 10.00 | 0.17 |  |
| **M** |  |  |  |  |  |
| M0 | 211 | 93.78 | 5962.00 | 99.22 | <0.0001 |
| MX | 1 | 0.44 | 11.00 | 0.18 |  |
| M1 | 10 | 4.44 | 26.00 | 0.43 |  |
| Unknown | 3 | 1.33 | 10.00 | 0.17 |  |
| **Chemotherapy** |  |  |  |  |  |
| Neoadjuvant | 40 | 17.78 | 625 | 10.40 | <0.0001 |
| Adjuvant | 78 | 34.67 | 1272 | 21.17 |  |
| None | 92 | 40.89 | 3952 | 65.77 |  |
| Missing | 15 | 6.67 | 160 | 2.66 |  |
| **Hormone therapy** |  |  |  |  |  |
| Yes | 90 | 40.00 | 3123 | 51.97 | <0.0001 |
| No | 119 | 52.89 | 2705 | 45.02 |  |
| Unknown | 16 | 7.11 | 181 | 3.01 |  |
| **Radiation therapy** |  |  |  |  |  |
| Yes | 109 | 48.44 | 2963 | 49.31 | 0.0649 |
| No | 96 | 42.67 | 2732 | 45.47 |  |
| Unknown | 20 | 8.89 | 314 | 5.23 |  |
| **Adjuvant therapy** |  |  |  |  |  |
| Yes | 78 | 35.94 | 1272 | 21.42 | <0.0001 |
| No | 132 | 60.83 | 4577 | 77.07 |  |
| Unknown | 15 | 6.91 | 160 | 2.69 |  |
|  | | | | | |

Abbreviations: AA, African-American; EA, European-American; T, tumor size; N, lymph node metastasis; M, distant metastasis.

**P* values were calculated using the x^2^ test.
