# Supplementary material for: Distinctions in Breast Tumor Recurrence Patterns Post-Therapy among Racially Distinct Populations
Source: PLoS One. 2017 Jan 13;12(1):e0170095. doi: 10.1371/journal.pone.0170095 (PMC5234824; doi:10.1371/journal.pone.0170095)
Supplement: S2 Table — (DOCX) [file pone.0170095.s003.docx]

**S2 Table. NH breast cancer patients’ demographics, clinico-pathological and treatment characteristics compared between AA and EA with recurrence.**

|  | **AA (n=49)** | | **EA (n=166)** | |  |
| --- | --- | --- | --- | --- | --- |
| **Clinical characteristic** | n | % | n | % | ***P*** |
| **Menopausal status** |  |  |  |  |  |
| <48 | 25 | 51.02 | 59.00 | 35.54 | 0.1452 |
| 48-55 | 6 | 12.24 | 33.00 | 19.88 |  |
| >55 | 18 | 36.73 | 74 | 44.58 |  |
| **Nuclear grade** |  |  |  |  |  |
| 1 | 2 | 4.08 | 21.00 | 12.65 | 0.29 |
| 2 | 17 | 34.69 | 62.00 | 37.35 |  |
| 3 | 26 | 53.06 | 73.00 | 43.98 |  |
| Missing | 4 | 8.16 | 10.00 | 6.02 |  |
| **Nottingham grade** |  |  |  |  |  |
| 1 | 3 | 6.12 | 10.00 | 6.02 | 0.4099 |
| 2 | 9 | 18.37 | 22.00 | 13.25 |  |
| 3 | 10 | 20.41 | 23.00 | 13.86 |  |
| Missing | 27 | 55.10 | 111.00 | 66.87 |  |
| **Stage** |  |  |  |  |  |
| 0 | 4 | 8.16 | 18.00 | 10.84 | 0.474 |
| I | 19 | 38.78 | 52.00 | 31.33 |  |
| II | 16 | 32.65 | 60.00 | 36.14 |  |
| III | 7 | 14.29 | 25.00 | 15.06 |  |
| IV | 2 | 4.08 | 6.00 | 3.61 |  |
| Missing/unknown | 1 | 2.04 | 5.00 | 3.01 |  |
| **Nodal status** |  |  |  |  |  |
| Positive | 15 | 30.61 | 62.00 | 37.35 | 0.5266 |
| Negative | 26 | 53.06 | 75.00 | 45.18 |  |
| Missing/unknown | 8 | 16.33 | 29.00 | 17.47 |  |
| **TNM Staging** |  |  |  |  |  |
| **T** |  |  |  |  |  |
| T0 | 0 | 0.00 | 2.00 | 1.20 | 0.422 |
| TX | 0 | 0.00 | 2.00 | 1.20 |  |
| Tis | 4 | 8.16 | 18.00 | 10.84 |  |
| T1 | 28 | 57.14 | 67.00 | 40.36 |  |
| T2 | 11 | 22.45 | 54.00 | 32.53 |  |
| T3 | 1 | 2.04 | 12.00 | 7.23 |  |
| T4 | 4 | 8.16 | 9.00 | 5.42 |  |
| Unknown | 1 | 2.04 | 2.00 | 1.20 |  |
| **N** |  |  |  |  |  |
| N0 | 31 | 63.27 | 101.00 | 60.84 | 0.7911 |
| NX | 0 | 0.00 | 1.00 | 0.60 |  |
| N1 | 12 | 24.49 | 44.00 | 26.51 |  |
| N2 | 4 | 8.16 | 13.00 | 7.83 |  |
| N3 | 1 | 2.04 | 4.00 | 2.41 |  |
| Unknown | 1 | 2.04 | 3.00 | 1.81 |  |
| **M** |  |  |  |  |  |
| M0 | 46 | 93.88 | 157.00 | 94.58 | 0.7855 |
| MX | 0 | 0.00 | 1.00 | 0.60 |  |
| M1 | 2 | 4.08 | 6.00 | 3.61 |  |
| Unknown | 1 | 2.04 | 2.00 | 1.20 |  |
| **Chemotherapy** |  |  |  |  |  |
| Neoadjuvant | 7 | 14.29 | 32 | 19.28 | 1 |
| Adjuvant | 18 | 36.73 | 58 | 34.94 |  |
| None | 20 | 40.82 | 66 | 39.76 |  |
| Missing | 4 | 8.16 | 10 | 6.02 |  |
| **Hormone therapy** |  |  |  |  |  |
| Yes | 17 | 34.69 | 71 | 42.77 | 0.6007 |
| No | 28 | 57.14 | 84 | 50.60 |  |
| Unknown | 4 | 8.16 | 11 | 6.63 |  |
| **Radiation therapy** |  |  |  |  |  |
| Yes | 24 | 48.98 | 81 | 48.80 | 0.9122 |
| No | 20 | 40.82 | 71 | 42.77 |  |
| Unknown | 5 | 10.20 | 14 | 8.43 |  |
| **Adjuvant therapy** |  |  |  |  |  |
| Yes | 18 | 38.30 | 58 | 36.25 | 0.3446 |
| No | 27 | 57.45 | 98 | 61.25 |  |
| Unknown | 4 | 8.51 | 10 | 6.25 |  |

Abbreviations: AA, African-American; EA, European-American; T, tumor size; N, lymph node metastasis; M, distant metastasis.

**P* values were calculated using the x^2^ test.
